# A549 as an In Vitro Model to Evaluate the Impact of Microplastics in the Air

**DOI:** 10.3390/biology12091243

**Published:** 2023-09-15

**Authors:** Chman Shahzadi, Alessandra Di Serafino, Eleonora Aruffo, Alessandra Mascitelli, Piero Di Carlo

**Affiliations:** 1 Center of Advanced Studies and Technology (CAST), University of “G. d’ Annunzio” Chieti Pescara, 66100 Chieti, Italy; chman.shahzadi@iusspavia.it (C.S.); alessandra.diserafino@unich.it (A.D.S.); eleonora.aruffo@unich.it (E.A.); piero.dicarlo@unich.it (P.D.C.); 2 University School for Advanced Studies IUSS Pavia, 27100 Pavia, Italy; 3 Department of Advanced Technologies in Medicine and Dentistry, University of “G. d’ Annunzio” Chieti Pescara, 66100 Chieti, Italy

**Keywords:** nanoplastics, polystyrene, A549, alveolar epithelial cells, air pollutants

## Abstract

**Simple Summary:**

This review article addresses the introduction and mechanism of action concerning the potential health effects of air-born microplastics on lung epithelial cell line A549. Micro- and nanoplastics are air-born pollutants, are inhaled, and cause lung toxicity by becoming internalized in cells, inducing genotoxicity, oxidative stress, immunomodulation, and morphological changes in A549 cells. These microplastics have the potential to accumulate in cells and trigger inflammation thereby contributing to the development of lung diseases. Numerous studies have investigated various aspects of micro- and nanoplastics on lung epithelial cells; however, the precise mechanism by which they initiate, or progress, lung toxicity is still subject to investigation. Nonetheless, the findings of previous studies strongly suggested that microplastics cannot be underestimated as insignificant air pollutants due to their potential to accumulate not only in the lungs but also in other organs such as the brain. This review article compiles studies on the effects of polystyrene microplastics on lung epithelial cells, highlighting existing research gaps, and providing suggestions for further research in this area.

**Abstract:**

Airborne microplastics raise significant concerns due to their potential health impacts. Having a small size, larger surface area, and penetrative ability into the biological system, makes them hazardous to health. This review article compiles various studies investigating the mechanism of action of polystyrene micro- and nanoplastics affecting lung epithelial cells A549. These inhalable microplastics damage the respiratory system, by triggering a proinflammatory environment, genotoxicity, oxidative stress, morphological changes, and cytotoxic accumulation in A549 cells. PS-NP lung toxicity depends on various factors such as size, surface modifications, concentration, charge, and zeta potential. However, cellular uptake and cytotoxicity mechanisms depend on the cell type. For A549 cells, PS-NPs are responsible for energy imbalance by mitochondrial dysfunction, oxidative stress-mediated cytotoxicity, immunomodulation, and apoptosis. Additionally, PS-NPs have the ability to traverse the placental barrier, posing a risk to offspring. Despite the advancements, the precise mechanisms underlying how prolonged exposure to PS-NPs leads to the development and progression of lung diseases have unclear points, necessitating further investigations to unravel the root cause. This review also sheds light on data gaps, inconsistencies in PS-Nos research, and provides recommendations for further research in this field.

## 1. Introduction

### 1.1. Micro- and Nanoplastics

The use of plastics in the world is rapidly increasing daily, and more than 380 million tons of plastics are produced yearly [1]. Despite this, most of the resulting plastic waste generated is still not treated appropriately, especially in developing countries. Global plastic recovery remains very low due to economic and social factors, and the widespread use of plastics also introduces serious consequences related to environmental pollution worldwide [2,3]. Increasing production, low recycling rates, and very low biodegradation rates make plastic materials hard to decompose and they remain in the environment for a long time. The different aging processes of plastic, like ultraviolet radiation, the physical–chemical, and biological process, lead to the formation of plastic particles in the environment in the size of centimeters, micrometers, and nanometers. These particles spread in land, water, and air, causing severe problems for humans and ecosystems. Small particles easily escape into the air and generate health complications for organisms breathing in air polluted by plastic [3].

Airborne microplastics can be of various types such as polystyrene (PS), polyethylene (PE), polypropylene (PP), polyvinyl chloride (PVC), and polyethylene terephthalate (PET). Their presence and distribution in the air depend upon various factors such as vertical gradient, sedimentation of particles, and meteorological and geographical factors.

Studies have demonstrated the characteristics of microplastics collected from various cities, such as Hamburg, Germany [4], Wenzhou City, China [5], and Beijing, China, which demonstrates that the collection of microplastics from the environment depends upon their size; studies have reported collections of MPs from the atmosphere tens of microns in size but recent studies have also reported particles and fibers of micro- and nanosize. The authors of [4] reported that 60% of MPs in Hamburg were >63 µm, while in Beijing, 80% of MPs were <20 µm [6].

Fibers, fragments, and granules are the dominant forms of MPs in the air. The size and shape of MPs determine their inhalation accessibility and potential toxicity, as some fibers become entangled and retained in gut tissues for longer periods of time and cause toxicity [7]. MPs in the air can also act as potential vectors for other environmental substances like DDT and hexachlorobenzene [8].

Microplastic distribution in indoor and outdoor atmospheres is different; it depends upon the sampling height [9]. Indoor MP concentration is observed as being higher (3–15 particles/m^3^) than outdoor concentrations (0.2–0.8 particles/m^3^) and rain can wash out MPs from the atmosphere. Rain also seems to be the factor responsible for the deposition of MPs [10]. Microplastics distribute in air and water according to their size, with larger airborne particles tending to sediment down. While in the marine environment, microplastics are present mainly on the surface and their concentration reaches 0 at 5 m depth of the water [11].

Air pollution resulting from microplastics and nanoplastics is a significant concern that cannot be overlooked. These minuscule particles, characterized by their small dimensions and extensive specific surface area, exhibit a notable propensity to infiltrate biological systems, giving rise to severe health implications. Microplastics (MPs) are characterized by having diameters smaller than 5 mm [12,13]. MPs could be fragmented by weathering to form even smaller plastic particles referred to as nanoplastics (NPs), with sizes below 100 nm and correspondingly large specific surface areas. This means that airborne NPs can form associations with environmental organic contaminants, and change their size, charge, and surface area which after penetrating into organs can induce toxicity with mechanisms different than pristine nanoplastics [14,15]. After inhalation, cellular toxicity is size-dependent as nanoplastics have a greater ability to penetrate and become internalized in biological systems than microplastics [16].

Observational researchers have reported breathing difficulties and intestinal inflammation in occupational workers exposed to microplastic particles and fibers [17]. Accidental occupational exposure to manufactured nanomaterials also affects the individuals in dose-dependent manners [18].

Research indicates that after absorption, MPs cross the epithelial barrier, reach the bloodstream, and distribute throughout the body. These MPs are found in human colon samples, feces, the placenta, and blood fields [19]. The MP amount in the feces of patients with inflammatory bowel disease is found to be 1.5-fold more than normal [19]. PS-NPs exhibit the propensity to also cross the blood–brain barrier (BBB) and accumulate in the brain leading to neurotoxic manifestations, as elucidated by Jung et al. (2020). Additionally, these PS-NPs have been found to significantly reduce the viability of mixed neuronal cells [20]. Yin et al. [21] studied PS-MP effects on chicken brain cells. They observed the onset of cerebral hemorrhage, pyroptosis induction, the generation of microthrombi, and the loss of Purkinje cells. Shan et al. [22] observed a significant number of PS-NPs crossed the blood–brain barrier (BBB) in rats in a dose-dependent manner and accumulated in brain cells. PS-NPs were also found in microglia, where they induced microglia activation and inflicted damage upon neurons. 

The vulnerability to cellular accumulation and toxicity of PS-NPs largely depends upon cell type. To investigate the adverse effects of PS-NPs in the lungs, A549 cells are excellent candidates to investigate toxicity. 

The study of NPs, especially in marine and freshwater ecosystems, has recently been recognized as a huge environmental global concern [23,24]. However, airborne microplastics and nanoplastics were not studied extensively for their toxic effects on inhaling organisms. Recently the scientific community has started to focus on the pollution status and ecotoxicological effects of NPs in the atmosphere and soil ecosystems. Unfortunately, the role of NPs especially in the atmosphere is still poorly understood. It has been widely observed that MPs can also reach far and remote areas, thanks to atmospheric transport processes. For example, traces of microplastics were found in sediments of remote locations on the top of Mount Everest and deep in the Mariana Trench [25]. Moreover, NPs, due to their extremely small diameter, high tissue affinity, and high adsorption capacity, can be easily inhaled by humans and other terrestrial animals. Their accumulation can lead to health effects, such as oxidative damage, inflammation, and even carcinogenicity [26,27], as well as biological responses in respiratory mucosa and lung tissue. However, the toxicity of NPs to lung cells remains mostly unexplored [15], although it has been shown that exposure to airborne pollutants may be responsible for aggravating several respiratory diseases. The link between air pollution together with lung malignancy [28], as well as increased morbidity and mortality linked to respiratory and cardiovascular causes, have been highlighted in several epidemiological studies [29,30]. 

In this systematic review, we collected and organized studies related to the toxic effects of polystyrene nanoparticles on A549 cells. This review aims to rationally combine all research findings that exist in the literature on this topic. This review will assess the findings and controversies in the results of various studies addressing the toxic impacts of PS nanoparticles on lung epithelial cells. 

### 1.2. A549

The lung carcinoma cell line A549 was isolated for the first time in 1973 from a pulmonary adenocarcinoma [31]. The A549 cell line represents a steady model for human alveolar type II pulmonary epithelium and has been widely used for studying toxicology, pharmacology, lung injury, and metabolic processing of lung tissue in vitro [32,33]. Since the A549 cell line closely mimics the human respiratory epithelium, it is an ideal and valuable tool for screening the ability of NPs to translocate through the pulmonary epithelium and to study this phenomenon in detail [15]. 

This review aims to underline the presence of MPs and NPs not only in water and soil ecosystems but also in the atmosphere, as well as their impact on the human respiratory tract through the A549 cell line. The results of this systematic review shed light on how deep is the gap in the evaluation of the effects of MPs and NPs on human health.

## 2. Methods 

### Search Strategy and Study Selection 

We systematically reviewed the literature on the potential effects of exposure to endocrine-disrupting chemicals (EDCs). We performed our search in March 2023, using the advanced search builder of the PubMed database, Google Scholar, Scopus, and Web of Science. We filtered hits by selecting articles published over the last 13 years (from 1 January 2010 to 31 January 2023), written in English, and excluding reviews. 

Our search included two main concepts: 1. polystyrene nanoparticles and 2. A549 cell line. A free search through titles and abstracts was added to obtain the latest articles. Hence, we added the following keywords for concept 1: polystyrene nano plastics, polystyrene nanoparticles, plastic nanoparticles, PNP, and PNPs. Keywords for concept 2 included A549 lung tumor cell, epithelial A549 lung, human lung carcinoma cell*, A549, A549 lung tumor cell*, epithelial A549 lung, human lung carcinoma cell*, human lung carcinoma, and human lung carcinoma. 

**Figure 1:**
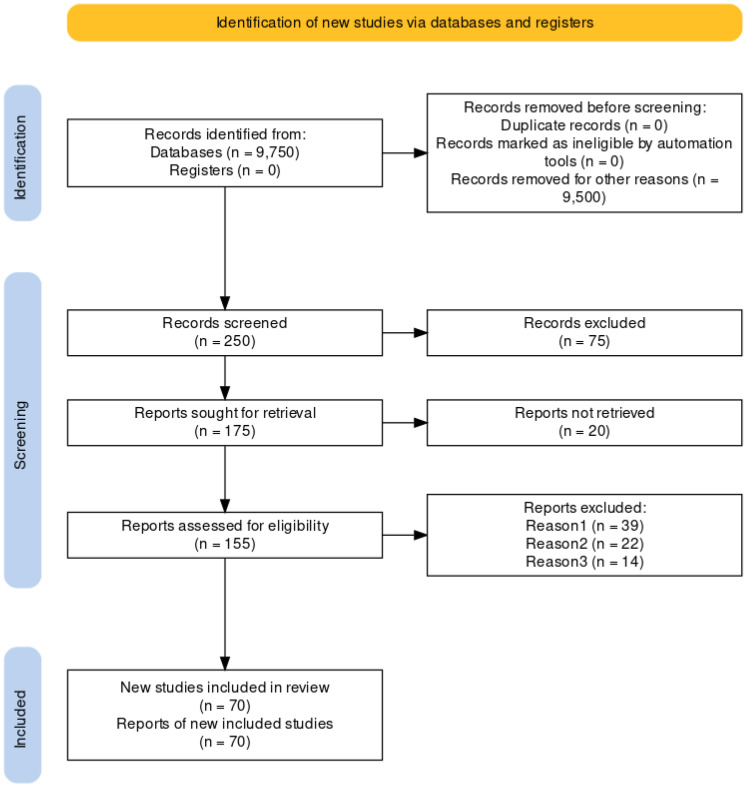
Study identification and selection process [34].

Two independent investigators extracted articles and determined eligibility. A third investigator resolved potential discrepancies. Our study selection process is depicted in Figure 1. First, we searched for studies related to PNPs (concept 1). After, we searched for articles on A549 (concept 2).

In our final screening, we excluded studies for three reasons: (1) they were not related to mammals (e.g., zebrafish); (2) they did not study toxic effects from PNP exposures; (3) they did not use A549 as a model. 

Internalization of PS particles is the first step that leads to cellular toxicity, and various processes are involved in the mechanism of internalization. Processes of PS toxicity investigated so far are included in this review, but further research is needed to fully understand the toxicity mechanism of PS particles which will lead to the development of treatment strategies to deal with complications initiated by air-born PS particles. We also included here suggestions for further research.

## 3. Results

**Figure 2:**
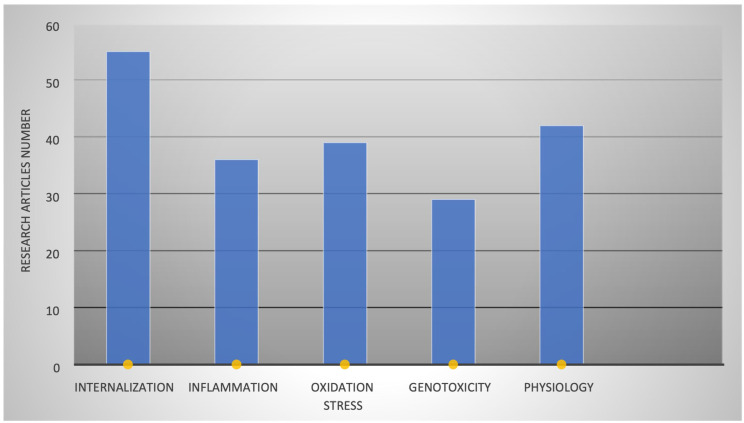
Number of research articles which covered the following aspects of PS-NPs: internalization of PS-NPs, inflammation, oxidative stress and genotoxicity, and change in the physiology of A549 cells because of PS-NP exposure.

PS-NP pulmonary toxicity is directly proportional to its internalization in cells, which depends upon the physiology of particles such as size, charge, zeta potential, and shape. After internalization, they induce inflammation, oxidative stress, and cell genotoxicity. Various studies have reported these findings using PS-NPs of diverse physical properties. Figure 2 illustrates the count of investigations conducted on A549 cells to explore these processes. Through thorough searches on Google Scholar, PubMed, Scopus, and Web of Science, we classified articles based on their examination of how PS-NPs impact various cellular processes. These investigations delved into the effects simultaneously. Consequently, we organized the articles according to their respective research focus and outcomes, presenting the information graphically in Figure 2.

### 3.1. PS-NP Types and Effects on Various Organs 

**Figure 3:**
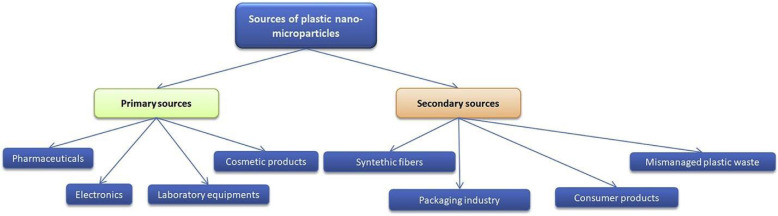
Types of PS-NPs and their sources [35].

PS-NPs can be primary or secondary in terms of their source. Primary PS particles are produced directly by industry and include cosmetics, paints, drugs, and plastic electronics, while secondary particles are generated from the disintegration of primary particles as a result of weathering, synthetic fibers, packaging materials, and improper plastic waste management (Figure 3). 

**Figure 4:**
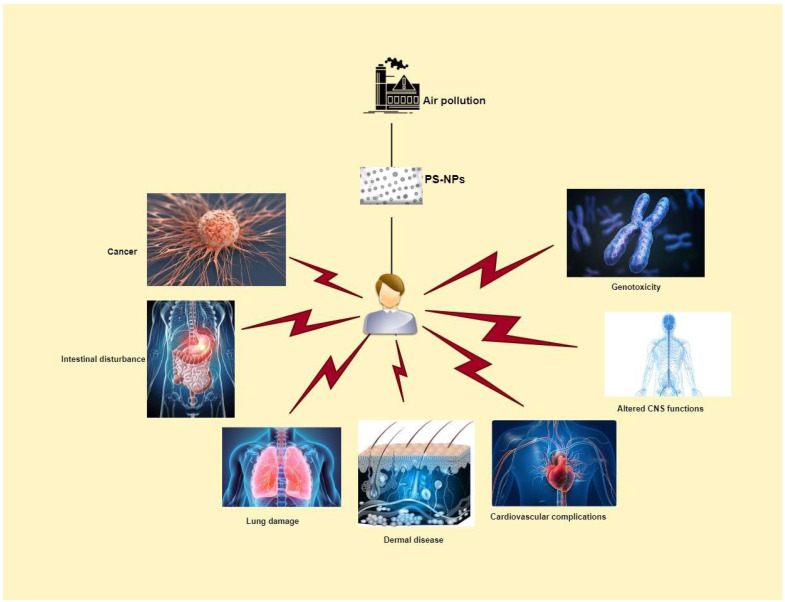
The diseases caused by air-born polystyrene particles in humans (images are taken from quizlet.com (accessed on 9 July 2023), istockphoto.com (accessed on 9 July 2023) and istockphoto.com (accessed on 9 July 2023).

PS-NP exposure is responsible for pulmonary toxicity, intestinal disturbance, carcinogenesis, dermal cell impairment, cardiovascular complications, alteration of CNS functions, and genotoxicity [36,37,38]. These disorders are the result of complex mechanisms of action of PS-NPs in respective cells (Figure 4). 

### 3.2. Physiochemical Properties Effects on PS-NP Internalization 

Studies suggest PS microplastics and nanoplastics size, charge, surface modifications, level, and time of exposure determine their toxicity level towards lungs after inhalation [37].

#### 3.2.1. Size of PS Particles Affecting Toxicity 

The size of PS particles defines their cellular uptake and toxicity., The authors of [15] investigated the cytotoxic effects of polystyrene nanoparticles (PS-NPs) on the A549 cell line representative of human lung epithelial cells. They considered PS-NPs with different diameters, 25 nm (PS-NP25) and 70 nm (PS-NP70), to evaluate viability and changes in the cell cycle and apoptosis and the transcription and expression of some important genes and proteins. 

They observed that PS-NP25 entered the cytoplasm of A549 cells more readily, in agreement with the conclusion of Debbage and Jaschke, that small particle size facilitates nanoparticle cell uptake [39]. The Xu’s group hypothesized that PS-NPs are internalized by nonspecific phagocytosis, and when A549 cells become saturated by internalized PS-NP70 nanoparticles, a release process may be activated, as shown in previous studies [27,40]. 

As far as cell viability is concerned, it is conditioned by PS-NPs in a dose-dependent manner; for example, PS-NP25 at near-lethal concentration slightly stimulated A549 cell viability, however low concentration PS-NP70 did not affect cell viability. Nevertheless, the pro-apoptotic protein expression levels of BAX, caspase-8, caspase-9, DR5, and cytochrome c, which are closely associated with initiating cell apoptosis, were up-regulated in PS-NP treated cells. In addition to this, transcription levels of pro-inflammatory cytokines and inflammation-related factors such as IL-6, IL-8, NF-κb, and TNF-α genes were up-regulated in PS-NP-treated cells. As a result, it was hypothesized that the inflammatory responses triggered by PS-NPs could disrupt the integrity of A549 cell membranes, potentially leading to subsequent cell necrosis.

Kihara et al. observed membrane disruption of A549 cells caused by PS smaller than 20 nm but not by bigger 200 nm PS-NPs [41]. They suggested membrane disruption does not significantly contribute to cytotoxicity, and there is the possibility that PS-NPs are indirectly responsible for membrane disruption. In rare cases, small particles (20 nm) were found in the proximity of chromosomes when there was no nuclear membrane around, like during mitosis; however, a significant accumulation of particles in that vicinity was not detected.

#### 3.2.2. Surface Modifications Effects on Toxicity 

Shi et al. [42] examined the cytotoxic impacts of PS-MPs of varying sizes and surface modifications. Their investigation specifically focused on the internalization of amino-modified and carboxy-modified polystyrene particles.

They observed more intracellular accumulation of PS-NH2 and PS-COOH than fundamental particles. Their findings were consistent with those of He et al. [43], who observed that modified polystyrene particles show more internalization compared to pristine PS-NPs in HepG2 cells. Surface functionalization increases the ionic interaction of PS-NPs with cell membranes [44]. PS-NH2 beads attach with cells strongly by electrostatic forces increasing internalization and toxicity while plain PS-NPs attach with cell membranes with acid-base attraction, van der Waals forces, and electrostatic forces [45]. Evaluating adverse effects of PS-NP genotoxicity is the main toxicological endpoint, as it is associated with carcinogenesis and other genetical pathologies. 

Shi, Wang et al. [42] observed various dose-dependent genotoxic and cytotoxic changes in A549 cells caused by 80 nm plain and surface-functionalized PS-NPs. However, PSNH2 caused more severe cytotoxicity in A549 cells than PS. This cytotoxicity was associated with mitochondrial damage leading to apoptotic cell death. As mitochondria are responsible for ROS balance, the damage in mitochondria will lead to ROS burst, further leading to oxidative damage in lipids, proteins, and DNA. Size surface modifications and time duration of PS-NP exposure defined the damage to A549 cells: smaller size particles are more capable of causing an excess in the oxidative stress, cell internalization, and cytotoxicity, while amino-modified PS-NH2 caused the greatest oxidation damage to cells in line with cell viability and genotoxicity. 

Shi et al. [46] studied the internalization, genotoxicity, and cytotoxicity of PS at different diameters (2 μm, 24 nm, and 80 nm) and surface modifications (carboxy and amino-functionalized polystyrene, pristine 25 polystyrene) in human A549 cells. 

Previous studies suggested that not only the size, physiochemical properties, and surface modifications of PS-NPs are responsible for toxicity, but also the interactions with different components present in receiving medium fields [42]. They measured the zeta potential of PS-NPs based on the size distribution and studied the internalization process of PS-NPs, which largely depends upon the size of the particle and the fate of internalized particle. The large surface area and special physical and chemical properties of PS-NPs provide convenience for their interaction with cell membranes, leading to internalization. In their experiment, they observed that 80 nm PS-NPs assemble in A549 cells more than PS-MPs, which suggested smaller nano-sized plastics are more readily taken up by the A549 cells, consistent with previous work. PS-NP accumulation in cells was not observed gradually increasing via increasing the concentration in a dose-dependent manner, as they observed a slight decrease in accumulation with an increasing dose. They suggested this was due to the PS-NP release process [15]. 

In conclusion, this study assesses the toxicological effects of PS-NPs on lung cells showing that the crucial factors are their size, internalization, intracellular concentration, and subcellular organelle response. Globally enhanced genotoxicity and cytotoxicity were observed due to nanoscale plastic particles’ easy uptake and intracellular accumulation and oxidative stress due to lysosomal leakage and apoptosis mediated by mitochondrial damage. 

Jeon et al. studied the role of surface charge in cellular uptake in A549 cells. They observed a 10–15% uptake of positively charged particles and a 6–7% uptake of negatively charged particles. They observed a positive correlation between the zeta potential and cellular uptake. The higher the zeta potential, the higher the cellular uptake [47]. 

**Figure 5:**
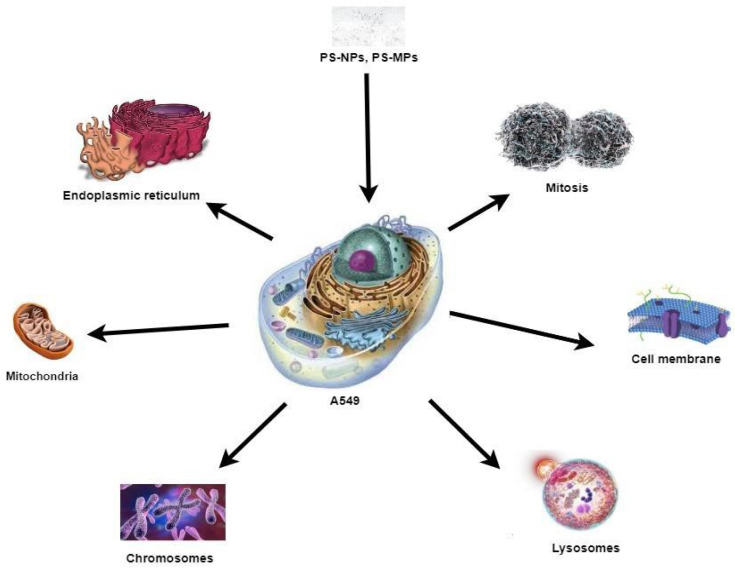
PS-NPs affect different organelles of A549 cells (images of organelles were taken from nigerianscholars.com (accessed on 9 July 2023), formative.com (accessed on 9 July 2023), quizlet.com and ck12.org (accessed on 9 July 2023).

After internalization into A549 cells, PS-NPs move to various cellular organelles and accumulate there [48]. Figure 5 represents the most affected organelles and cellular processes of A549 cells due to PS-NP exposure. 

## 4. Effects of PS-NPs on Human Health 

### 4.1. Genotoxicity 

Yang et al. [49] studied potential pulmonary toxicity and the underlying mechanism of PS-NPs on lung epithelial cells. They used 7.5, 15, and 30 μg/cm^2^ concentrations of PS-NPs to assess the alteration in their genetic expression through microarrays. They observed that PS-NPs induce inflammation by destabilizing the redox balance and significantly decrease cell viability, triggering apoptotic pathways leading to cell death. They used 7.5 μg/cm^3^ and 30 μg/cm^3^ concentrations of PS-NPs (40 nm) and observed the extensively altered gene expression of the 755 genes. These genetic alterations were enriched in oxidoreductase activity and transcription regulation. This caused redox imbalance and altered transcription regulations, which was suggested as the possible cause of the pathogenesis of respiratory disease. They performed functional analysis to assess the potential mechanism: altered genetic expression was observed in genes centralized in the Hippo signaling pathway, TNF signaling pathway, Pl3K-Akt signaling pathway, and other cancer-related pathways. They hypothesized that cell death induced by redox imbalance might be the central mechanism of PS-NP-induced lung injury. 

Zhang et al. studied PS-NP-induced genetic alterations in different cell lines [50]. They observed a change in 1812 gene expression in two treated groups (high dose, low dose) with a consistent trend: 700 genes were changed only in the low dose group, and they suggested these genes played a key role in sublethal damage, which is reversible, and cells repair to a normal state when exposure is removed. They also studied genes that changed both in low- and high-dose groups for GO (Gene Ontology) [51] and KEGG (Kyoto Encyclopedia of Genes and Genomes) analysis [52]. They observed that a quarter of GO terms exhibited a link with oxidative stress, such as the oxidoreduction coenzyme metabolic process, oxidoreductase activity, and oxidation–reduction process. In their experiments, the KEGG pathway has clear links with oxidative stress. To understand the link between oxidative stress and genotoxicity, they identified 10 TFs regulating DEGs using the DAVID website. They found that nine out of ten TFs were associated with oxidative stress with a ranking of FOXO3 > STAT3 > FOXO4 > BACH1 > SOX9 > GATA2 > GATA6 > NFE2 > PBX1, according to the relevance score based on the Genecards database. FOXO3 plays a central role in various biological processes, especially oxidative stress. Moreover, they constructed the regulatory network between nine TFs and oxidative stress-associated DEGs to visualize their interaction. They also incorporated a rat lung injury model induced by PS-NPs from the GO database into their experiment of DEGs retrieved from the Invitro study. They took 16 candidate genes from the microarray analysis of rat lung tissues. After intersecting these findings with genes of the TFmRNA regulatory network, they identified the TNFRSF12A gene (also known as Fn14). The latter is involved in various pathological processes such as oxidative stress, angiogenesis, inflammation, carcinogenesis, proliferation, and death. 

The evidence has suggested that Fn14 combined with TWEAK increase the oxidative stress by NADPH oxidase in macrophages [53]. Recently, another study validated this evidence and suggested that Fn14 is involved in the development of the respiratory disease [54]. 

### 4.2. Endocytosis and Exocytosis of PS in A549 Cells 

PS-NP internalization depends on time, dose, cell type, and particle size. The higher the dose and longer the time, the more the cellular uptake of PS will be. Liu et al. [55] studied 50 nm and 100 nm PS-NP internalization by A549 cells. They observed that 50 nm PS was ingested more in cells than 100 nm, although it depends on the time of exposure and concentration. 100 nm PS particles are distributed mainly in lysosomes in the cell, while 50 nm particles were found around the cell membrane. The energy-dependent cellular intake process requires more energy to intake larger particles [56]. Excretion of larger particles was observed to be more difficult than in the smaller ones. They observed no considerable excretion of 100 nm PS particles, while 50% of 50 nm particles were excreted out in 6 h. The lysosomes of the A549 cells mediated this efflux. 

Varela et al. [57] studied size-dependent uptake of PS-NPs in A549 cells. They examined 20 nm, 40 nm, and 100 nm sizes. Surprisingly, they found out that 40 nm particles have the highest uptake, followed by 100 nm and 20 nm. They explained that larger particles can attach with cells with higher van der Waals force, as the attraction is directly proportional to the surface and diameter of spheres. They explained the 40 nm particles’ faster intake by assuming that the cells use different kinetics of the endocytosis mechanism to internalize the particles. 

Dos Santos et al. [58] studied PS particle uptake at different temperatures by cells to explore whether it is an active or passive process. For this reason, they incubated cell lines at 37 °C and 4 °C and exposed these to 40 nm and 200 nm PS-NPs. Strong endocytosis inhibition was observed in the 4 °C group. An average 90% uptake was inhibited for 40 nm particles, and 70% uptake was inhibited for 200 nm particles, proving this cellular uptake of PS-NPs is an energy-dependent process. 

Sipos et al. [59] observed that the PS particle uptake in A549 cells is through the micropinocytosis process. They performed an egress experiment and observed 90% PS egress in 24 h. PS-NPs were distributed in the cytoplasm. PS-NP egress by increasing cytosolic Ca^2+^ level by lysosomes was observed in different cells, but in contrast, the A549 cells’ Ca^2+^ levels increase over PSNP exposure with no alteration in the kinetics of PS-NP egress over a 24 h period. They assumed that this is because PS-NP might be leaked into the cytoplast from lysosomes and may not be available for lysosomal exocytosis. After 24 h exposure, they found a relatively low concentration of PS-NPs in lysosomes, and most PS-NPs were found in lysotracker green-negative vesicles. This explains why an increase in Ca^2+^ failed to speed up the egress of PS from lysosomes. 

### 4.3. Oxidation Stress Caused by PS 

Oxidation stress assay revealed a significantly decreased expression of antioxidant enzymes, like HO-1 and NQO1, and increased oxidative stress. This increased oxidative stress in the PS-NP-treated group was suggested as the primary contributing factor for the pathogenesis of lung disease [59]. PS-NPs were found to induce inflammation and oxidative stress, resulting in damage to lung cells. This was evident through the release of LDH, signifying the compromised membrane integrity of lung epithelial cells. This observation aligns with prior studies, which suggested that increased oxidative stress may lead to a loss of membrane integrity.

Sipos et al. [59] studied the effects of PS-NPs on mitochondrial function using TMRM dye, considered the most sensitive method. They observed no significant decrease in TMRM fluorescent intensity after exposure to PS particles. They also assessed mitochondrial membrane potential using a novel mitophagy dye and observed minimal or no change in mitochondrial functions. They suggested that lysosomes are more sensitive than mitochondria to PS exposure. 

### 4.4. Morphological Changes in A549 Cells on PS-NPs Exposure 

The authors of [60] studied morphological changes induced in A549 cells by PS (1 µm and 10 µm) at 60, 72, and 96 h of exposure. While the A549 in the control group showed their typical appearance, with the puffy cells in close contact with little individual variations between cells, cells exposed for 72 h to 1 µm particles seemed to be no longer in close contact with their neighboring cells. This is in line with the observed loss of the epithelial cell tight junction in the normal bronchial cell line. After the exposure to 10 µm particles, the cells showed a similar morphology; in fact, uniformly shaped cells lost cell-to-cell contact at a rapidly growing stage and adopted many features of motile cells such as attaching to lamellipodia, followed by the appearance of microspikes and filopodia. Loss of cell-to-cell contact is the first morphological change observed in cells, which turns into a dramatic change in morphology after increasing exposure time to 96 h, with no chance to resemble the original cells. As the appearance of cytoskeletal features occurs, these cells obtained the ability to move perhaps at different sites. They also observed substantial inhibition in the proliferation of cells exposed to PS particles. 

### 4.5. Inflammation Caused by PS 

It was observed that exposure to PS-NPs caused the secretion of interleukin and the recruitment of inflammatory cells, and activated the immune system [61]. Previous studies have suggested that this local inflammation can amplify cell death and tissue injuries at different sites, both in the lungs and reproductive system. 

Brown et al. studied PS-NP immunomodulatory effects and observed that 202 nm and 535 nm PS did not cause any PMN (polymorphonuclear leukocyte) influx in cells, while 64 nm PS caused a considerable influx of PMN. However, inflammation was observed in all cell groups; thus, they suggested that the smaller size and larger surface area might be responsible for driving inflammation. Less than 100 nm respirable particles behave differently than larger ones, and this might be the cause of inflammogenicity in the lungs. They observed Ca^2+^ influx in PS-NP-treated cells after their treatment with thapsigargin. This rapid influx allows Ca^2+^ to act as a signaling molecule; this imbalance in calcium homeostasis might activate transcription factors, leading to the expression of proinflammatory genes. They treated A549 cells with 64 nm PS-NPs and observed changes in the expression of IL-8, a potent neutrophil chemoattractant. After a 2 h exposure, significant IL-8 expression was observed, meanwhile, after 4 h this expression was lower than at the 2 h exposure level. This would explain the inflammation observed in the PS-NP-treated cells. They hypothesized that the PS-NPs deposit on epithelium cells and trigger oxidative stress, stimulating IL-8 gene expression. The latter then leads to neutrophil recruitment causing inflammation. This study also observed that prolonged PS-NP exposure also affected the repair mechanism, leading to lung disease and tissue damage [62]. 

### 4.6. PS Effects on Epithelial–Mesenchymal Transition (EMT) 

Halimu et al. evaluated the possible risks of pulmonary fibrosis after PS-NP exposure. They investigated the ability of PS-NPs to cause the epithelial to mesenchymal transition (EMT) of A549 cells that is a prelude to lung fibrosis [37]. EMT is a key step for human epithelial cell fibrosis, via ER stress and NOX4-mediated mitochondrial dysfunction. They observed that small-sized positive surface-charged PS-NPs exerted the most severe effects. They also observed a negative correlation between ROS production and cell viability. Moreover, they found NOX4-specific siRNA inhibited intracellular ROS production, and significantly improved viability of PS-NP-treated A549 cells in medium to high concentration. A possible explanation they suggested is that the ROS-related oxidative stress may have caused the cytotoxic effect of PSNPs. It is speculated that EMT is induced through NOX4-activated ROS formation by PS-NPs, and then subsequent stimulation of the mitochondrial respiratory chain triggers ROS release. The amplified ROS signals caused different levels of mitochondrial dysfunctions which may define the different outcomes of cell viability. This study observed that medium to high concentrations of PS-NPs decreased cell viability via damaging the inner mitochondrial membrane through depressing respiratory capacity, loss of ATP production, and decreased mitochondrial membrane potential (Δψm). According to the findings of [63], the NOX4-derived ROS production may lead to the dissipation of Δψm accompanied by an ROS burst, which generally is called ROS-induced ROS release (RIRR). This RIRR may irreversibly destroy the mitochondria, leading to further EMT enhancement, induced by PS-NPs in lung A549 cells. The authors of [64] concluded that the low concentration of PS-NPs can increase glycolytic ATP production to compensate for the deficit in mitochondrial oxidative phosphorylation-derived ATP production. However, a high concentration of PS-NPs decreases the glycolytic ATP synthesis, proving that irreversibly damaged mitochondria cannot maintain the cellular repair and energy metabolism compensation in lung A549 cells. ROS generated by PS-NPs induce misfolding in ER and destabilize ER homeostasis [65]. Senft and Ze’ev observed that the NOX4 gene silencing inhibited the PS-NP-induced EMT and decreased the ER stress markers as ATF4 and BiP. They concluded that ER stress, induced by NOX4 and related to ROS production, is somehow responsible for EMT in PS-NPs exposed to A549 cells. 

### 4.7. Accumulation of PS-NPs in the Brain 

PS-NPs are likely to cross the blood–brain barrier and accumulate in the brain to cause neurotoxicity. PS-NPs (100 nm) can significantly reduce the viability of mixed neuronal cells as elevated levels of apoptosis markers and cleaved caspase-3 were observed. Yin et al. studied PS-NP effects on chicken brain cells and they observed cerebral hemorrhage, generation of microthrombi, and loss of Purkinje cells [21]. Intracerebral hemorrhage leads to strong immune cell infiltration, activating signaling pathways to induce pyroptosis. PS-NP exposure caused mitochondrial dysfunction and activated AMPK signaling. They concluded PS-NP exposure leads to intracerebral injury, triggering cerebellar tissue inflammation and pyroptosis. 

Shan et al. investigated the ability of 50 nm PS-NPs to cross the blood–brain barrier (BBB) [22]. They observed a significant increase in the permeability of PS-NPs across the BBB in dose-dependent manners and accumulation in mouse brain cells. PS-NPs were found in microglia, where they induced microglia activation and neuron damage. The in vitro study on immortalized human cerebral microvascular endothelial cells (hCMEC/D3) revealed that PS-NPs penetrated cells, generated ROS, activated nuclear factor kappa-B, secreted tumor necrotic factor-α, and caused necroptosis of cells [22]. 

Upon PS-NP- exposure, cultured neural stem cells and mice in developmental stages demonstrated altered neural stem cell (NSCs) functions.

Maternal exposure to PS-NPs during gestation and lactation caused alteration in neural cell composition and brain histology in progeny. High concentration of PS-NP exposure resulted in abnormal brain development and neurophysiological and cognitive deficits in mice’s gender-specific manners [66]. In another study [67] the authors gave oral doses of PS-NPs to maternal mice and later they were found in the alimentary tract, brain, placenta, and uterus in maternal mice and infiltrated into the thalamus of the fetus. As a result, they observed anxiety-like behavior and oxidative injury in progeny.

Another experiment was performed by [68], during which they gave oral doses of PS-MPs to mice and observed the accumulation of PS-MPs in the brain’s microglial cells. They followed their experiment with differential treatment of PS-MPs in human microglial cells and observed changes in immune responses, cellular morphology, and microglial apoptosis induced by phagocytosis of 0.2 µ PS-MPs. 

### 4.8. Cytotoxicity Studies of PS Particles 

The authors of [46] combined PS-NPs (100 nm) with phthalates esters (PAEs) to assess the cytotoxic effects on A549. Although plastic particles and PAEs coexist extensively in various compartments of the environment, their combined toxic effects on organisms are largely unknown [69], with even less known about co-toxicity on human health. 

In the studies [15,48], the uptake of PS-NPs in A549 was observed. PS-NPs exhibited cytotoxicity at concentrations higher than 200 µg, but the combined exposure to PS-NPs and PAEs on A549 was still greater than NP-only treatments. This indicates that co-exposure is hazardous as well. Their study reduced the free concentration of PAEs in the cell medium by the co-presence of PS-NPs. Exposure to PS-NPs (200 ng) determined an enhanced amount of intracellular ROS, equivalent to the ROS levels expressed in co-exposure with PAEs. This suggests that there is a correlation between intracellular accumulation and bioavailability. 

From the quantification of SOD, CAT, GSH-Px activities, and concentrations of the lipid peroxidation product, malondialdehyde (MDA), a correlation was found between the concentration of PS-NPs and levels of oxidative stress. Although during the combined exposure to PAEs, oxidative stress was higher, the PS-NP concentration was greater. The inflammatory response was evaluated through transcriptional studies. Exposure to PS-NPs and PAEs results in a significant up-regulation of IL-1β, IL-6, IL-8, and TNF-α, in agreement with what was reported by Xu et al. (2019). These inflammatory reactions could be responsible for the destruction of A549 cell membrane integrities, resulting in a necrosis [15,70]. It has been reported that when inhaled matter enters the respiratory system, in addition to ROS, to recruit inflammatory cells, cytokines are also secreted by airway epithelium. These local inflammatory responses can be amplified, giving rise to subsequent systemic inflammation, and resulting in COPD and asthma [71,72]. 

Table 1 shows the summary of studies where different sizes of PS particles were used to assess their effects on A549 cells. The resultant disorders and alterations in functions of A549 cells are also listed. Observation of similar results of various studies using the same parameters validated certainty in the body of evidence.

#### Is One Cell Culture Model Enough to Assess the Microplastic Toxicity?

Static cell culture models are not entirely accurate in assessing microplastic internalization because of buoyancy issues. Plastics vary in terms of density as PS has a density of 1 g/cm^−3^, while PP, PE, PU, and PEST have densities <1 g/cm^−3^. If microplastic density is lower than the medium, in cell culture models microplastics will float on the surface of the medium and will not entirely incorporate with cells which are mostly adherent with the bottom of the culture flask [74]. This way, the toxicity value will not be assessed accurately. More microplastic internalization was observed when static cell culture models were compared with microfluidic models in the case of PS particles, showing that conventional cell culture might not reflect the actual internalization and underestimate the value of cellular toxicity caused by microplastics [75].

While the A549 cell culture model, Raw264.7 and THP-1 cells representing macrophages are commonly employed to evaluate lung cytotoxicity, it has been demonstrated that co-culture models are more effective in replicating the real exposure environment. Lehner et al. conducted research involving co-culture models, specifically utilizing human intestinal epithelial cell lines Caco-2 and HT29-MXT-E12 in combination, as well as human blood monocyte-derived dendritic cells and macrophages in co-culture models in comparison to single cell culture models [76]. A549 and THP1 co-culture study in comparison to single cell cultures revealed the different amount of cell uptake and cytotoxicity expression level validating the importance of co-cultures in microplastic toxicity research [77].

To depict the effects of microplastics on the lungs more accurately in a real-life context, the use of an air–liquid interface (ALI) model is recommended. This model allows for the assessment of airborne pollutant exposure in both single-cell culture and co-exposure models, providing a more realistic approach. Several commercially available ALI models, such as EpeAirwar, OncoCilAirTM, and MucilAirTM [78], are accessible for research purposes [78]. 

## 5. Discussion

The presence of micro- and nanoplastic pollution in freshwater and marine environments and their toxicological effects have been widely reported. On the contrary, very few assessments have been completed to measure the impact of air-born nanoplastics on terrestrial animals, particularly on the respiratory system. Consequently, MP and NP interactions with human lung cells, especially in terms of toxicity or cellular uptake, are still widely debated and far from conclusive [15,28]. 

PS-NPs are one of the most representative nanoplastic particles in the scientific literature, as they can be found in a wide range of industries. The global demand for polystyrene (PS) and expanded PS has increased incredibly, with an estimated value of USD 49 billion by 2025 (Market Report Coverage, 2017). Synthetically, PS-NPs can be synthesized in a wide range of sizes. They are also commercially available in a fluorescent core-labeled format, which determines a huge trend of studies aimed to track them in living cells [15,79].

Day by day, an increasing magnitude of plastic pollution is an environmental threat, as many plastic particles have been detected in coral reefs, streams, rivers, oceans, and in the air, and PS has been proven to be one of the main components of atmospheric particulate matter (PM) pollution [25,80]. Public concern about plastic pollution is growing exponentially.

Plastics tend to degrade into smaller particles through weathering and environmental factors; with time and with further degradation, these particles escape in air, and add to the PM pollution in the air.

Most of the (microplastics) MPs detected in the atmospheric environment are 50 μm to 5 mm in size [25]. However, this is due to the limits of detection methods for MPs; in fact, in natural atmospheric environments, it is logical to suppose that large MP particles are thought to be further degraded into smaller particles with nanometer size [15], also known as nanoplastics (NPs). As highlighted by the research group of Xu [15], “if humans have inhaled sufficient amounts of NP the intactness and functions of human alveolar type II pulmonary epithelium would be destroyed, followed by local inflammatory reactions and respiratory mucosal barrier damage, even causing an increased probability of opportunistic infections by airborne pathogenic microorganisms”. The authors of [15] observed that there is a specific path in which A549 cells internalize PS-NPs and that this depends on particle size and time of exposure. PS-NPs with smaller sizes showed more potential for internalization than larger-sized particles. PS-NPs were able to disturb gene expression, resulting in inflammatory responses and launching apoptosis pathways.

The authors of [81] concluded that when A549 cells were co-exposed to polystyrene NPs and PAEs (DEHP and DBP), they exhibited changes in cell viability, oxidative stress, and inflammatory reaction. Moreover, the combined toxic effects of NPs and PAEs on A549 cells were influenced by the exposure levels of NPs and the properties of compounds. Their study provides the first detailed insight into the effect of NPs on the cytotoxicity of PAEs in A549 cells expanding our knowledge of the potential risk assessment of NPs and combined pollution on human health, which is the more likely scenario in our daily lives as we are continuously co-exposed to a combination of several air pollutants. 

Studies observed that oxidative stress is the main factor that initiates cell damage. As [59] suggested, the oxidative stress induced by PS-NPs is the main factor responsible for the pathogenesis of lung disease. Oxidative stress also leads to various disorders, like DNA damage, loss of membrane integrity, and inflammation. 

It is undeniable that MPs and especially NPs can cause definite damage and functional disturbance to human and mammalian respiratory systems. At the same time, few studies are currently trying to assess the risk factors associated with PS-NP uptake.

## 6. Conclusions

Nanoplastic pollution is raising concern because it reaches the most remote locations on earth by air and water transport. Its accumulation magnitude is so high that it is considered an emergent geomaterial with potential toxicological consequences. 

Studies have indicated the deleterious effects of PS-NPs on lung epithelial cells (A549); after being inhaled, they induce lung toxicity by triggering oxidative stress, immunomodulation, and genetic alterations. 

PS-NPs with a size of 25 nm or smaller pose a greater long-term inhalation risk. Among the 70 studies conducted, only 6 indicated that PS-NPs might not be a significant concern, while the remaining 64 reported adverse effects. These conflicting findings can be attributed to various factors, including differences in particle characteristics such as size and shape (fibers, fragments, and spherical particles), surface modifications, and variations in study design. To address these controversies, it is crucial to adopt more realistic research approaches, such as using models that mimic the conditions of the airway, like ALI (air–liquid interphase) models, cell co-cultures, and examining the combined effects of PS-NP exposure with other environmental pollutants. Furthermore, research should focus on assessing how PS-NP exposure exacerbates pre-existing lung diseases. It is also essential to determine the concentration of PS-NPs in the air, and how it differs geographically. Estimation of average daily exposure levels for individuals and conducting comprehensive analyses would help to estimate to risk posed to the health of humans by PS-NPs.

The exact mechanism of PS-NPs to generate toxicity, and their impacts on various organs are not entirely known. More research is necessary to assess the lung damage caused by air-born plastic particles.

## 7. Suggestions for Future Research 

Interaction of PS-NPs with other air pollutants could potentially lead to alteration in their physical and chemical properties changing their toxicity mechanism for health. In addition to the purchased samples used, samples should also be obtained from the real environment of the production factory or PS-NP-rich air and their toxicity should be observed and compared with purchased samples to validate the previous findings on synthesized PS-NPs.

In addition to increasing the concentration of PS-NPs, the exposure time should be increased with the concentration present in a real environment, to provide a more accurate insight into the effects of PS-NPs mimicking real exposure. 

The effects of co-exposure of PS-NPs with other environmental pollutants on physical and chemical properties of PS-NPs and effects of alteration in their toxicity should also be assessed, as in real-life, toxicity is caused by co-exposure with other environmental pollutants. 

The metastasis of adverse effects from the lungs to other parts of the body caused by lengthy exposure to PS-NPs needs to be assessed to obtain a wider picture of PS-NP toxicity. 

Methods to clear PS-NPs from the environment should be found, either by attaching some ligand or by weathering and decomposition chemically, to invent sprays to dispose of these PSNPs from the indoor work environment, to eliminate the indoor and at the later stages also the outdoor PS-NP pollution. 

More extensive knowledge about the exact mechanism of PS-NP toxicity would lead to research on the development of therapeutic strategies to relieve the adverse effects of PS-NPs on health. 

In microplastic research concerning lung toxicity, it is advisable to embrace novel approach methods (NAMs). These include the utilization of air–liquid interphase (ALI) models and the co-culture of A549 cells with other cell types. These innovative techniques will help to provide a means of obtaining accurate results while also offering alternatives to animal-based methods.

## Figures and Tables

**Table 1:** Summary of studies investigated the impacts of PS-NPs on A549 cells.

Polymer Type	PS Size	Biological Model	Results	References
PS-NPs	25 nm, 70 nm	A549 cells	Upregulated inflammatory gene expression, lost cell membrane integrity, necrosis	[15]
PS-NPs	20 nm	A549 cells	Membrane disruption	[41]
PS-NPs	40 nm	BEAS-2B and HPAEpiC	Altered genetic expressions, redox imbalance-mediated inflammation leading to apoptosis	[49]
PS-NPs	50 nm, 100 nm	A549 cells	Size- and charge-dependent cellular internalization of PS-NPs, uptake is energy-dependent which decreases at low temperature	[55]
PS-NPs		A549 cells	Oxidation stress	[59]
PS-NPs	1 µm and 10 µm	A549	Cells lose close contact with neighboring cells and grow apart. Generation of Cytoskeletal features and ability to move at distant places, inhibition in proliferation	[60]
PS-NPs	64 nm	A549	Oxidative stress leading to IL-8 expression, stimulating neutrophil recruitment and inflammation.	[62]
PS-NPs	20 nm, 50 nm	A549	Endoplasmic reticulum stress, mitochondrial dysfunction, oxidative stress.	[37]
PS-NPs, PS-NH2, PS- COOH	80 nm, 2 μm	A549	Size-dependent internalization of PS particles, more internalization of surface-functionalized PS particles.	[42]
PS-NPs	116 nm, 152 nm	A549	Transport of PR-NPs in cell is assisted by microtubules and transported by lysosomes	[48]
PS-NPs	200 nm	A549	Zetapotential of PS shows negative correlation with toxicity.	[73]
PS-NPs, Carboxylated PS-NPs	20 nm, 40 nm, 100 nm	A549	Cellular uptake is size-dependent but 40 nm internalized more than 20 nm	[57]
fluorophore-conjugated polystyrene nanoparticles (F-PLNPs)	82 nm	A549	Cellular internalization shows positive correlation with zeta potential, surface charge of PS impacts greatly on cellular uptake	[47]
PS-MPs	10 μm, 1 μm	A549	Decreased in metabolic activity and proliferation rate of cells, changed cell morphology	[60]
